# Imbalance of the CD226/TIGIT Immune Checkpoint Is Involved in the Pathogenesis of Primary Biliary Cholangitis

**DOI:** 10.3389/fimmu.2020.01619

**Published:** 2020-07-24

**Authors:** Chuiwen Deng, Wenli Li, Yunyun Fei, Li Wang, Yingying Chen, Xiaofeng Zeng, Fengchun Zhang, Yongzhe Li

**Affiliations:** ^1^Department of Rheumatology, National Clinical Research Center for Dermatologic and Immunologic Diseases (NCRC-DID), Peking Union Medical College Hospital, Chinese Academy of Medical Science and Peking Union Medical College, Beijing, China; ^2^Department of Rheumatology, China-Japan Friendship Hospital, Beijing, China; ^3^Department of Clinical Laboratory, Peking Union Medical College Hospital, Chinese Academy of Medical Science and Peking Union Medical College, Beijing, China

**Keywords:** primary biliary cholangitis, CD226, TIGIT, immune checkpoint, pathogenesis

## Abstract

The relationship between the cluster of differentiation 226 (CD226)/T cell Ig and ITIM domain (TIGIT) immune checkpoint and primary biliary cholangitis (PBC) pathogenesis is unknown. Herein, PBC patients (*n* = 42) showed significantly higher proportions of peripheral CD8^+^ T and CD4^+^ T cells expressing either CD226 or TIGIT than disease (*n* = 25) and healthy (*n* = 30) controls. The percentage of CD8^+^TIGIT^+^ T cell was negatively associated with total bilirubin, direct bilirubin, total bile acid, γ-glutamyl transpeptidase, and alkaline phosphatase, but positively correlated with platelet count; alkaline phosphatase was positively associated with the frequency of CD8^+^CD226^+^ T cell; and the CD226/TIGIT ratio of CD8^+^ T cell was positively associated with total bilirubin, direct bilirubin, total bile acid, γ-glutamyl transpeptidase, alkaline phosphatase, and aspartate aminotransferase to platelet ratio, but negatively correlated with albumin and platelet count. The effector function of CD8^+^CD226^+^ T cells was more robust than the CD8^+^CD226^−^ counterparts. CD226 blockade reduced CD107a^+^, IFN-γ^+^, and TNF-α^+^ proportions among CD8^+^CD226^+^ T cells, inhibiting CD8^+^ T cell proliferation. In conclusion, CD226/TIGIT immune checkpoint imbalance is involved in the pathogenesis of PBC. The CD226/TIGIT ratio of CD8^+^ T cell is a potential biomarker for evaluating the disease status and the prognosis of PBC patients. Moreover, CD8^+^CD226^+^ T cells represent a possible therapeutic target for PBC, and blocking CD226 could inhibit the activity of this cell subset *in vitro*.

## Introduction

Primary biliary cirrhosis (PBC) is a chronic non-suppurative destructive cholangitis, characterized by the progressive impairment of small and medium-sized intrahepatic bile ducts that leads to liver fibrosis and potential cirrhosis (1). Anti-mitochondrial autoantibodies are detected in most PBC patients and autoreactive lymphocyte infiltration is observed in their livers (2, 3). Although autoimmunity plays a vital role in the pathogenesis of PBC, biologic therapies targeting the immune system have not proven as successful as those directed at the bile ducts or fibrosis (4).

Immune checkpoints are one of the most important regulatory tools of the immune system. T-cell-related checkpoints have been shown to be involved in immune escape during malignancies (5). A recent study has indicated that the immune checkpoint is responsible for the continuous expansion of the abnormal immune response, making it a potential therapeutic target for PBC (6).

One immune checkpoint pathway that includes two receptor, cluster of differentiation 226 (CD226)/T cell Ig and ITIM domain (TIGIT), has been drawing increasing attention from researchers. TIGIT, a coinhibitory factor expressed on effector and memory T cells, transfers a negative signal into cells expressing this factor upon binding to its ligand CD155, mainly leading to the suppression of the immunological response. CD226 competitively binds the same ligand and plays the opposite role in regulating the immune system (7). The high frequency of TIGIT^+^ lymphocytes in cancer has been shown to be involved in the escape from immune surveillance (8). Similarly, the frequencies of TIGIT-expressing immune cells have been reported to be associated with the disease activities of autoimmune diseases (9–11). Interestingly, previous studies have shown that CD226 is also involved in autoimmunity dysfunction. Aberrant expression of peripheral CD226 has been observed in rheumatoid arthritis (12, 13), systemic lupus erythematosus (14, 15), and systemic sclerosis (16). In addition, a CD226 gene polymorphism (rs763361 C>T) has been shown to be associated with susceptibility to type 1 diabetes mellitus (17).

Although the functional competition relationship between TIGIT and CD226 has been established, only a few studies have explored the mechanism underlying their joint involvement in disease pathogenesis. The autoimmune disease studies published to date have mainly focused on one single receptor, i.e., either TIGIT or CD226.

The role that TIGIT and CD226 play in the pathogenesis of PBC is yet to be elucidated. Therefore, we performed this study to assess the proportional changes and clinical associations of the CD226/TIGIT immune checkpoint in T cells from PBC patients. Based on the observed correlations, we further characterized the alteration of T cell effector function and proliferation due to CD226 blockade to explore the potential of the CD226/TIGIT pathway as a therapeutic target.

## Materials and Methods

### Participants

Peripheral blood samples were obtained from participants at the Peking Union Medical College Hospital; 42 PBC patients who fulfilled the diagnosis criteria (18), and showed no other autoimmune or liver diseases were enrolled. The age- and sex- matched disease controls (DCs) group contained 25 patients with other chronic liver diseases (including 11 non-alcoholic steatohepatitis, 8 alcoholic steatohepatitis, and 6 autoimmune hepatitis cases), and the healthy controls (HCs) group included 30 apparently healthy participants. Liver function test results and immunoglobulin levels were collected from the participants. The aspartate aminotransferase to platelet ratio was calculated according to the following formula: [(glutamic oxaloacetic transaminase/upper limit of normal glutamic oxaloacetic transaminase level) × 100]/ platelet count (10^9^/L). The clinical data are summarized in Table 1. This study was approved by the Ethics Committee of the Peking Union Medical College Hospital; written informed consent was obtained from all participants.

**Table 1:** Characteristics of the enrolled participants.

	**Primary biliary cholangitis**	**Disease controls**	**Healthy controls**
	**(*n* = 42)**	**(*n* = 25)**	**(*n* = 30)**
Age (years old)	58 (30–76)	51 (27–69)	53 (25–67)
Female gender (n, %)	39, 92.9%	21, 84.0%	26, 86.7%
Alanine aminotransferase	29.5 (9.0–251.0)	46.0 (13.0–182.0)	12.0 (2.0–16.0)
Aspartate aminotransferase	41.0 (16.0–198.0)	24.0 (16.0–124.0)	19.0 (11.0–36.0)
Alkaline phosphatase	133.5 (55.0–909.0)	121.0 (53.0–708.0)	62.0 (30.0–89.0)
γ-glutamyl transpeptidase	69.5 (11.0–745.0)	57.0 (20.0–624.0)	17.0 (3.2–29.0)
Total bilirubin	14.0 (4.9–74.7)	11.3 (6.3–57.2)	7.0 (2.1–16.0)
Direct bilirubin	4.7 (1.4–44.2)	5.5 (1.9–35.0)	1.5 (0.6–5.4)
Total bile acid	9.70(1.4–436.9)^†^	8.8 (0.5–303.3)	2.7 (1.2–9.0)
Albumin	43.0 (27.0–48.0)	46.5 (41.0–50.0)	31.0 (23.0–50.0)
Immunoglobulin G	15.2 (8.5–33.2)^‡^	15.7 (10.4–24.5)	12.0 (8.8–16.7)
Immunoglobulin A	2.8 (0.3–5.4)^‡^	2.8 (1.6–3.2)	2.1 (0.5–3.9)
Immunoglobulin M	2.1 (0.6–8.5)^‡^	1.2 (0.7–2.0)	1.4 (0.5–2.3)
Platelet count	230 (51–305)*	242 (115–376)	ND
Aspartate aminotransferase to platelet ratio	0.7 (0.2–2.6)*	0.4 (0.2–1.0)	ND
Anti-mitochondrial antibody positive (n)	38	0	0
Anti-gp210 antibody positive (n)	11^§^	0	ND
Anti-sp100 antibody positive (n)	4^§^	0	ND

All the PBC patients were undergo ursodeoxycholic acid treatment only. Data were depicted as Median (Min, Max) otherwise. Reference interval: Alanine aminotransferase: 7–40 U/L; Aspartate aminotransferase: 13–35 U/L; Alkaline phosphatase: 35–100 U/L; γ-glutamyl transpeptidase: 7–45 U/L; Total bilirubin: 5.1–22.2 μmol/L; Direct bilirubin: 0–6.8 μmol/L; Total bile acid: <10 μmol/L; Albumin: 35–52 g/L; Immunoglobulin G: 7–17 g/L; Immunoglobulin A: 0.7–4 g/L; Immunoglobulin M: 0.4–2.3 g/L; Platelet: 100–350 × 10^9^/L; Anti-mitochondrial antibody: <1:80; Anti-gp210 antibody: Negative; Anti-sp100 antibody: Negative. Available in 39 PBC patients; Available in 32 PBC patients; *Available in 35 PBC patients; ^§^Available in 23 PBC patients. ND, not detected.

### Cell Preparation

Overnight fasting venous blood samples were collected from the study participants by venipuncture using sterile, EDTA-treated tubes (BD Biosciences, San Jose, USA). Peripheral blood mononuclear cells (PBMCs) were separated by density gradient centrifugation (Histopaque-1077; Sigma-Aldrich, Merck, Darmstadt, Germany).

### Monoclonal Antibodies for Flow Cytometry

The following monoclonal antibodies were used: anti-CD3-APC-H7 (SK7), anti-CD4-PerCP/Cy5.5 (RPA-T4), anti-CD8-PE-Cy7 (RPA-T8), anti-CD25-APC (3C7), anti-CD226-FITC (DX11), anti-CD107a-PE-CF594 (H4A3), anti-HLA-DR-APC (TU36), anti-TNF-α-APC (Mab11), and anti-IFN-γ-PerCP-Cy5.5 (B27) antibodies, purchased from BD Bioscience; anti-TIGIT-PE (MBSA43) and anti-TIGIT-APC (MBSA43) antibodies, purchased from ThermoFisher (Waltham, MA, USA); and anti-CD226-PerCP/Cy5.5 (11A8) antibodies, purchased from BioLegend (San Diego, California, USA).

Data were acquired using a FACSAria II (BD Biosciences) instrument, followed by analysis with FlowJo software version 7.6 (TreeStar, Ashland, OR, USA). A control antibody of the respective IgG isotype was included in all experiments.

### Cell Immunophenotypic Analysis

In order to measure the expression of surface markers CD226 and TIGIT on T cells, PBMCs were resuspended in PBS and then stained with anti-CD3, anti-CD4, anti-CD8, anti-CD25, anti-CD226, and anti-TIGIT antibodies. Non-regulatory T (Treg) CD4^+^ and CD8^+^ cells were initially identified using fluorochrome-conjugated anti-CD3, anti-CD4, anti-CD8, and anti-CD25 antibodies. The CD4^+^ and CD8^+^ T cells were then further subdivided into the TIGIT^+^, TIGIT^−^, CD226^+^, and CD226^−^ subsets by TIGIT or CD226 staining. Relative data are presented as the percentage of CD226^+^ or TIGIT^+^ cells among CD4^+^ or CD8^+^ T cells.

### Functional *in vitro* Assay

PBMCs were washed in PBS containing Ca^2+^ and resuspended in RPMI 1640 plus 10% fetal bovine serum. Leukocyte activation cocktail containing GolgiPlug (BD Biosciences) was added and the cells, which were then cultured at 37°C in a humidified atmosphere containing 5% CO_2_ for 4 h for their activation. Next, human leukocyte antigen-DR isotype (HLA-DR) was stained to determine the activation status of the T cells. For intracellular staining, the cells were subsequently fixed and permeabilized using the IntraSure Kit (BD Biosciences) and TNF-α and IFN-γ were then stained with the respective monoclonal antibodies.

### CD226 Blocking

In order to block CD226, PBMCs were washed and resuspended and an anti-CD226-FITC antibody, which can facilitate CD226 blocking, as well as the subsequent flow cytometry analysis, was then added, followed by incubation for 20 min. Next, leukocyte activation cocktail containing GolgiPlug was added for 4 h to activate the cells. CD3, CD4, CD8, CD107a, IFN-γ, and TNF-α were stained as described above to determine the functional and activation changes in T cells due to CD226 blocking.

To assess the proliferation, PBMCs were stained with 1 μM carboxyfluorescein diacetate succinimidyl ester (CFSE; Sigma-Aldrich) at 37°C for 15 min, and then washed and resuspended in RPMI 1640 medium containing 10% fetal bovine serum. These labeled PBMCs were incubated with mouse anti-human CD3 (5 μg/mL; BD Bioscience) and mouse anti-human CD28 (5 μg/mL; BD Bioscience) antibodies for 72 h at 37°C in a humidified atmosphere containing 5% CO_2_, until the surface markers CD3, CD4, and CD8 were stained; then, the cells were analyzed by flow cytometry. In order to avoid a quenching effect, most of the above-mentioned procedures were performed in the dark.

### Statistical Analysis

All data are presented as the means ± standard deviations, unless otherwise noted. The Kolmogorov-Smirnov test and Shapiro-Wilk test were used to analyze normality. A paired or unpaired *t*-test was used to compare the data from the two groups and Welch's correction was applied when the data had a non-normal distribution. Pearson' correlation or Spearman's rank correlation analysis was used for normally distributed or non-parametric variables, respectively. Statistical analyses were performed using GraphPad Prism Version 5.0 (GraphPad, San Diego, CA, USA).

## Results

### Phenotypic and Clinical Associations of CD226 and TIGIT on T Cells

**Figure 1:**
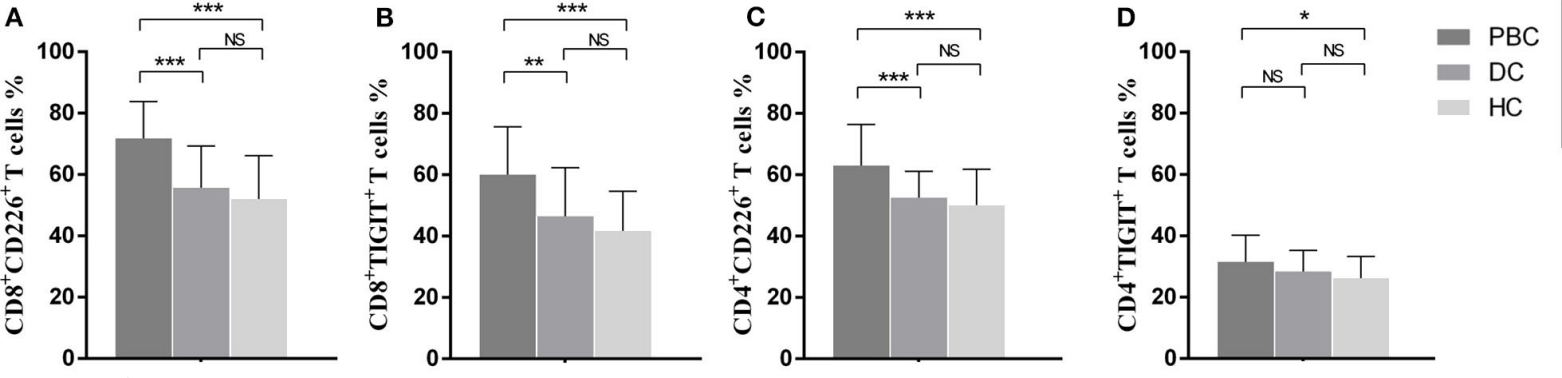
Frequencies of CD226- and TIGIT-positivity in peripheral T cells from PBC patients, disease controls, and healthy controls. Proportional comparison of the peripheral CD8^+^CD226^+^ T cells **(A)**, CD8^+^TIGIT^+^ T cells **(B)**, CD4^+^CD226^+^ T cells **(C)**, and CD4^+^TIGIT^+^ T cells **(D)** among groups. The data of each group are presented as the means ± standard deviations. *p < 0.05; **p < 0.01; ***p < 0.001.

Significantly increased frequency of CD8^+^CD226^+^T cells was found in PBC patients compared to that in the DCs (71.81 ± 11.99 vs. 55.67 ± 13.66, *p* < 0.001) and HCs (71.81 ± 11.99 vs. 52.04 ± 14.12, *p* < 0.001) (Figure 1A). The patients with PBC also showed a markedly increased percentage of CD8^+^TIGIT^+^ T cells than the DCs (60.0 ± 15.60 vs. 46.44 ± 15.85, *p* = 0.011) and HCs (60.0 ± 15.60 vs. 41.73 ± 12.92, *p* < 0.001) (Figure 1B).

With regard to the phenotypic analysis of CD4^+^T cells, the percentage of CD4^+^CD226^+^ T cells was significantly higher in PBC patients than in DCs (63.07 ± 13.30 vs. 52.55 ± 8.54, *p* < 0.001) and HCs (63.07 ± 13.30 vs. 50.10 ± 11.70, *p* < 0.001) (Figure 1C). When comparing the proportions of CD4^+^TGIT^+^ T cells among the groups, only the difference between the PBC patients and HCs was significant (31.50 ± 8.70 vs. 26.20 ± 7.10, *p* = 0.032) (Figure 1D).

In CD8^+^T cells, the frequency of TIGIT^+^ cells was negatively associated with total bilirubin (*r* = −0.38, *p* = 0.01), direct bilirubin (*r* = −0.43, *p* < 0.01), total bile acid (*r* = −0.35, *p* = 0.03), γ-glutamyl transpeptidase (*r* = −0.35, *p* = 0.02), and alkaline phosphatase (*r* = −0.39, *p* = 0.01), but positively correlated with platelet count (*r* = 0.38, *p* = 0.03). Moreover, alkaline phosphatase was positively associated with the proportion of CD8^+^CD226^+^ T cells (*r* = 0.37, *p* = 0.02). The clinical association observed between the proportion of TIGIT^+^ cells among the CD4^+^ T cells and that of CD226^+^ cells among the CD4^+^ T cells was not significant. No clinical association was observed in DCs and HCs.

### Clinical Associations of the CD226/TIGIT Ratio in PBC

As CD226 and TIGIT play diverse roles in the immune system, a value combing both might represent the real status of the entire immune system. Therefore, we introduced a CD226/TIGIT ratio, calculated as the proportion of CD226^+^ cells among CD8^+^T cells divided by that of TIGIT^+^ cells among CD8^+^T cells or the percentage of CD226^+^ cells among CD4^+^T cells divided by that of TIGIT^+^ cells among CD4^+^T cells. The CD226/TIGIT ratio of CD8^+^T cells was positively associated with total bilirubin (*r* = 0.31, *p* = 0.04), direct bilirubin (*r* = 0.35, *p* = 0.02), total bile acid (*r* = 0.47, *p* < 0.01), γ-glutamyl transpeptidase (*r* = 0.31, *p* = 0.04), alkaline phosphatase (*r* = 0.42, *p* < 0.01), and the aspartate aminotransferase to platelet ratio (*r* = 0.35, *p* = 0.04), but negatively correlated with albumin (*r* = −0.40, *p* = 0.02) and platelet count (*r* = −0.34, *p* = 0.04). No clinical association was found in DCs and HCs. As the CD8^+^ T cells showed important clinical associations in PBC, we decided to focus on these cells in subsequent experiments.

### Activation Status of T Cells in PBC

HLA-DR, a late T cell activation marker, was used to evaluate the activation status of T cells in PBC. The proportion of TIGIT was significantly increased in HLA-DR^+^CD8^+^ T cells compared with HLA-DR^−^CD8^+^ T cells (63.15 ± 17.39 vs. 42.6 ± 13.99, *p* < 0.01), which is in accordance with a previous report that the expression of TIGIT might be regulated by negative feedback from an over-activated immune response. Combing with the negative clinical correlation found in the frequency of CD8^+^TIGIT^+^ T cells and the CD226/TIGIT ratio of CD8^+^ T cells, TIGIT appears to play a protective role in the development of PBC.

### Influence of CD226 Blocking on T Cells

**Figure 2:**
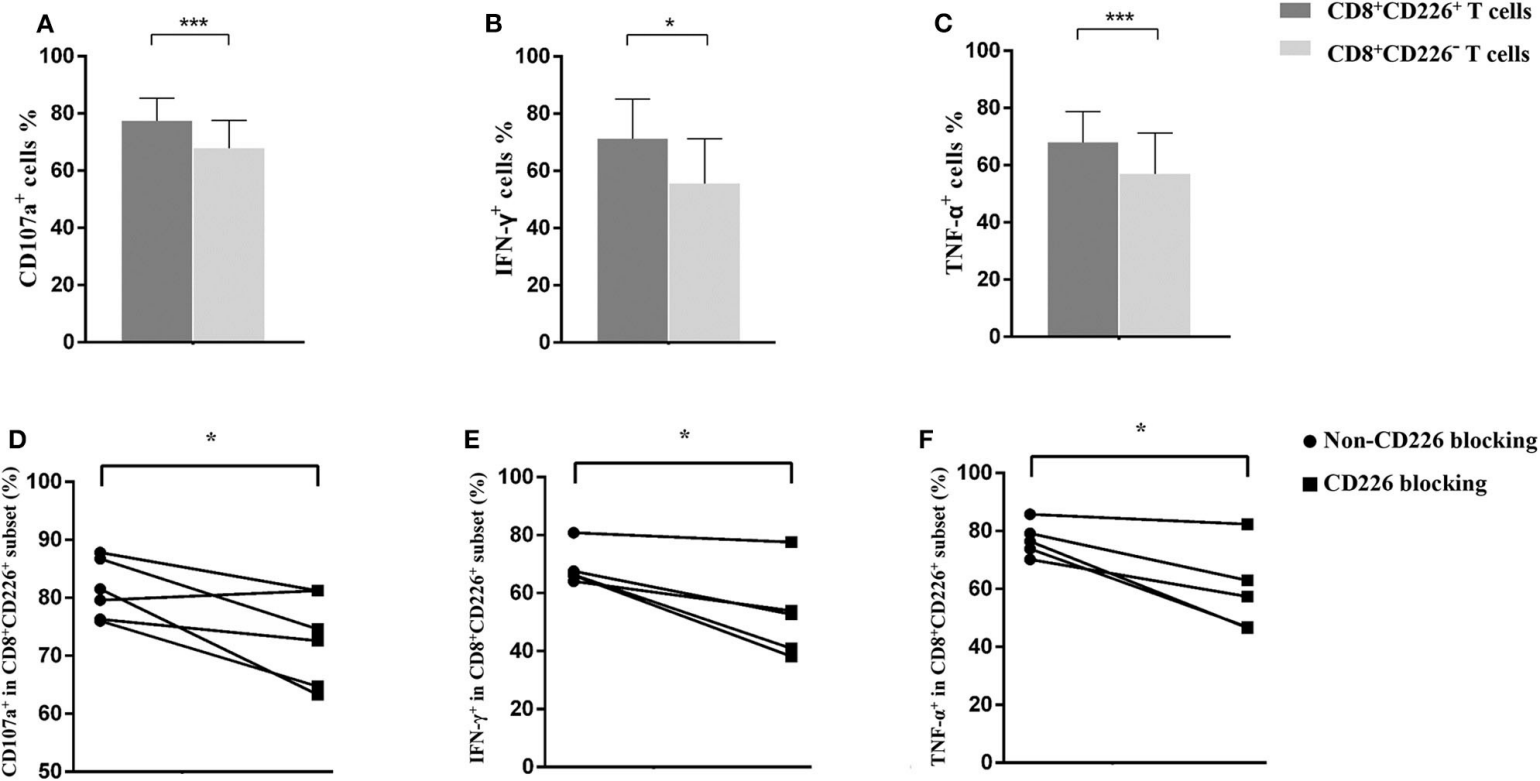
Influence of CD226 blocking on CD8^+^T cells from PBC patients. Comparison of the effector function between CD8^+^CD226^+^ T cells and their CD8^+^CD226^−^ counterparts by CD107a **(A)**, IFN-γ **(B)**, and TNF-α **(C)** staining. The functional alteration of CD8^+^ T cells by CD226 blocking in terms of CD107a **(D)**, IFN-γ **(E)**, and TNF-α **(F)** expression. The data of each group are presented as the means ± standard deviations. *p < 0.05; ***p < 0.001.

**Figure 3:**
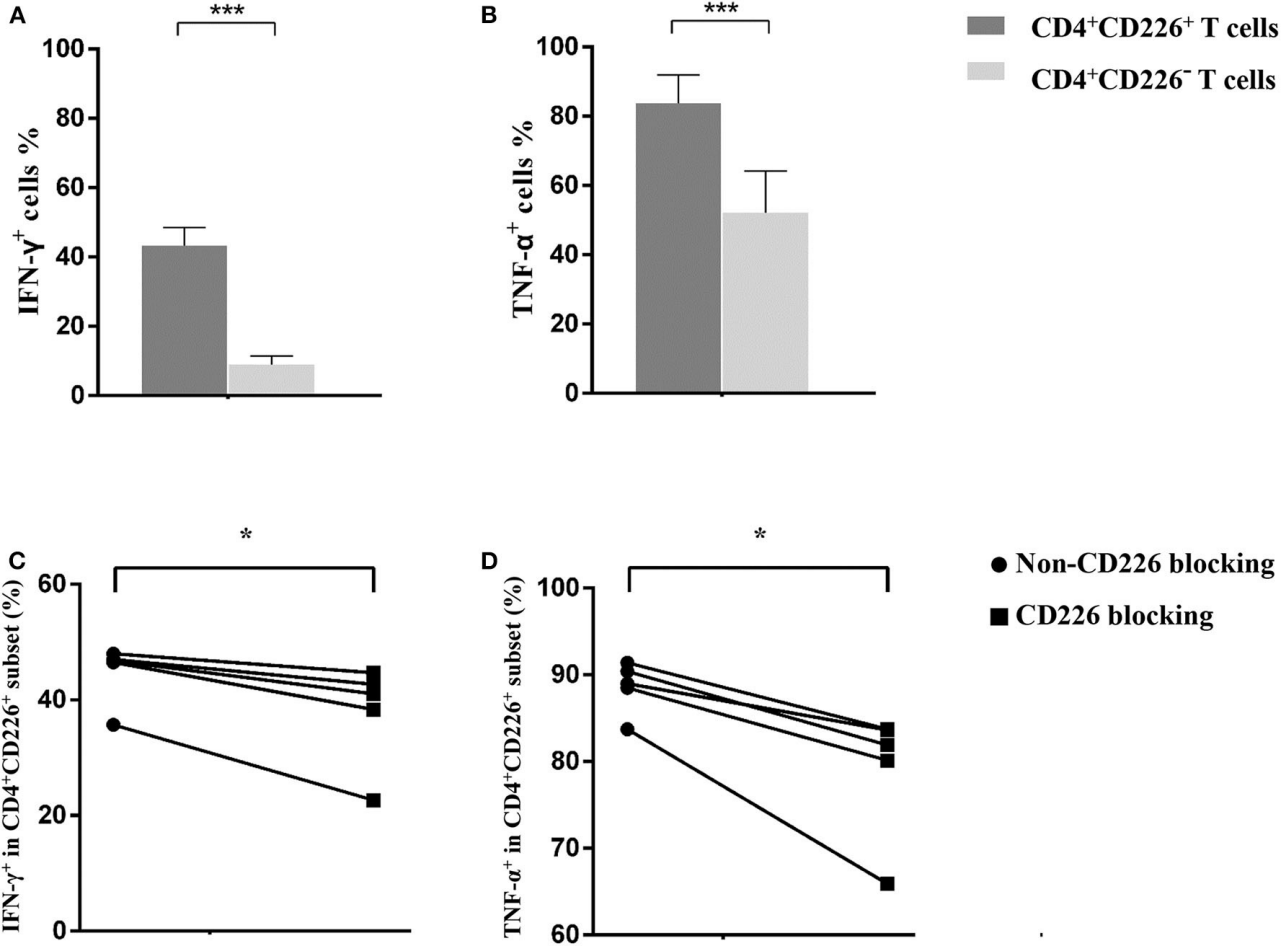
Influence of CD226 blocking on CD4^+^T cells from PBC patients. Comparison of the effector function between CD4^+^CD226^+^ T cells and their CD4^+^CD226^−^ counterparts by IFN-γ **(A)** and TNF-α **(B)** staining. The functional alteration of CD4^+^ T cells by CD226 blocking in terms of IFN-γ **(C)** and TNF-α **(D)** expression. The data of each group are presented as the means ± standard deviations. *p < 0.05; ***p < 0.001.

Based on the observed results, we further explored the elevated expression of CD226 and its relationship with disease deterioration. The effector function of T cells was evaluated by staining for CD107a, IFN-γ, and TNF-α, and results showed that CD226^+^ T cells were more robust than CD226^−^ counterparts (Figures 2A–C, 3A,B). Thus, we next examined whether reducing CD226 efficiency could alleviate T cell overactivity in PBC.

**Figure 4:**
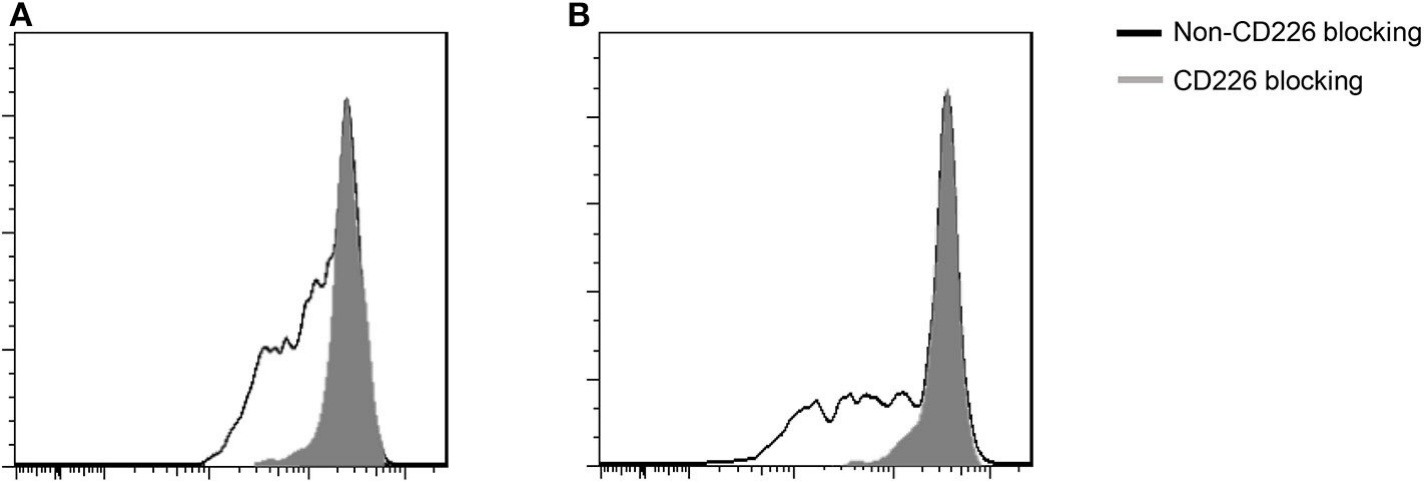
Influence of CD226 blocking on the proliferation of T cells from PBC patients. **(A)** CD8^+^ T cells; **(B)** CD4^+^ T cells.

Blocking CD226 on CD8^+^T cells from PBC patients resulted in significantly decreased frequencies of CD107a^+^ (81.12 ± 5.05 vs. 72.73 ± 7.75, *p* = 0.032; Figure 2D), IFN-γ^+^ (69.18 ± 6.73 vs. 52.86 ± 15.58, *p* = 0.024; Figure 2E), and TNF-α^+^ (76.42 ± 5.88 vs. 58.56 ± 14.71, *p* = 0.021; Figure 2F) cells among CD8^+^CD226^+^T cells, compared with non-blocked cells. Moreover, significantly reduced percentages of IFN-γ^+^ (45.0 ± 5.12 vs. 38.12 ± 8.82, *p* = 0.016; Figure 3C) and TNF-α^+^ (88.30 ± 2.97 vs. 78.74 ± 7.49, *p* = 0.011; Figure 3D) were also observed in CD226-blocked CD4^+^ T cells from PBC patients. The proliferation of CD226^+^ T cells also could be inhibited by CD226 blockade (Figure 4). In summary, our results imply that the CD226 blockade led to the suppression of the effector function and proliferation in both CD4^+^CD226^+^ T and CD8^+^CD226^+^ T cells in PBC patients.

## Discussion

Previous researches have confirmed that biologics targeting immune checkpoints, such as abatacept, which targets the CTLA-4 related immune checkpoint, have high potential for clinical application in treating autoimmune diseases (19). However, abatacept has not demonstrated any efficacy in PBC; no biochemical responses or improved clinical outcomes were observed during treatment (20), indicating that the excessive autoimmune response in PBC might be due to other immune checkpoints. In recent years, CD226/TIGIT, a novel immune checkpoint, was identified; it is considered as one of the most important brakes of the immune system (7, 21). To date, the CD226/TIGIT pathway has been explored and successfully applied in the clinical treatment of several diseases (21, 22). Previous genome-wide association studies also revealed an important link between the CD226/TIGIT immune checkpoint and autoimmune diseases (17); however, its relationship with the pathogenesis of PBC has not been illustrated. In this study, we explored the CD226/TIGIT pathway balance in PBC patients for the first time.

Comparing with the controls, increased frequencies of CD226 and TIGIT on T cells were observed in PBC patients. Interestingly, significant clinical associations between the CD226/TIGIT immune checkpoint and PBC disease status were found in CD8^+^ T cells, but not CD4^+^ T cells. As we know, CD8^+^ T cells represent a T cell subset that extensively infiltrate liver lesions in PBC. More importantly, CD8^+^ T cells in PBC have been reported to play an adoptive transfer role that could induce autoimmune cholangitis (23). Our results not only add to the growing evidence that CD8^+^ T cell abnormality plays a vital role in the pathogenesis of PBC, but also indicate a potential pathway by which the CD226/TIGIT immune checkpoint might be involved in the adoptive transfer of PBC by CD8^+^ T cells.

The main difference between our study and previous studies investigating TIGIT or CD226 in autoimmune diseases (9–15, 17) is that we simultaneously explored the T cells expired molecular, TIGIT, as well as its competitive receptor, CD226. Considering the opposite role of these checkpoint molecules play in the T cells activity, we introduced the CD226/TIGIT ratio value for the first time. Statistical analysis showed the CD226/TIGIT ratio of CD8^+^ T cells has more clinical correlations than that of CD8^+^CD226^+^ T cells and CD8^+^TIGIT^+^ T cells in PBC patients. These significant associations imply that an imbalance between CD226 and TIGIT exists in the CD8^+^ T cells of PBC patients, which could lead to liver damage when the proportion of CD226^+^ cells among the CD8^+^T cells is higher than that of TIGIT^+^ cells among the CD8^+^T cells, presenting as elevated digestive enzyme levels in serum and decreased ability of synthesizing liver proteins such as albumin. More importantly, the CD226/TIGIT ratio of CD8^+^T cells was positively correlated with the aspartate aminotransferase to platelet ratio, a prognostic factor of fibrosis, suggesting that this ratio could serve as a potential prognostic biomarker.

In PBC patients, the CD8^+^T cells expressing HLA-DR contained a higher frequency of TIGIT^+^ cells, which is in accordance with previous reports (9, 10) that the expression of TIGIT is regulated by negative feedback on activated cells. Therefore, TIGIT may serve as a protective factor in PBC, which is different from the role that TIGIT plays in malignancies (8). On the other hand, based on the observed correlation, the elevated frequency of CD8^+^CD226^+^ T cells may be a risk factor for PBC patients. Moreover, increased frequencies of IFN-γ- and TNF-α-producing cells were observed in CD8^+^CD226^+^ T cells, indicating that they possess a more robust effector function than CD8^+^CD226^−^ T cells. CD107a expressed on the membrane of lytic granules is transiently exposed to the extracellular side of the CD8^+^ T cells during degranulation, an important pathway by which CD8^+^ T cells exert their cytotoxicity. Previous studies have confirmed that CD107a not only indicates the occurrence of degranulation, but is also a sensitive marker for evaluating the activity of cytotoxic cells (24, 25). Higher percentages of CD107a^+^ cells among CD8^+^CD226^+^ T cells were observed in PBC patients, implying that they have greater cytotoxicity than their CD8^+^CD226^−^ counterparts. Taken together, these results confirm that CD8^+^CD226^+^ T cells are the dominant proinflammatory subsets in PBC.

A recent open-label study has shown that the biological agent abatacept lacks clinical efficacy in PBC, possibly because it could not reduce the frequency of terminally differentiated T cells, especially, CD8^+^T cells (20). Based on the multiple clinical associations and the functional difference between the subsets we observed, both CD226 blockade and enhancing the intrinsic inhibitory properties of TIGIT in PBC patients are potential therapeutic targets for inhibiting terminally differentiated T cells. We first attempted to block the CD226 pathway in PBMCs from PBC patients and found that CD226 blocking could not only suppress the proliferation of T cells, but also inhibit their cytotoxic function by decreasing the expression of CD107a and reducing the cytokine production of IFN-γ and TNF-α. With regard to enhancing the expression of TIGIT, we tried to use several commercial TIGIT antibodies as agonists for *in vitro* experiments; however, our initial results showed that the clones we used could not effectively enhance the inhibitory function of TIGIT (data not shown). Previous studies have shown that different anti-TIGIT antibody clones have diverse biological functions and that they can act as TIGIT-blocking antibodies, have agonistic activity on TIGIT, or have no influence on T cells (26). Therefore, further studies should attempt to generate agonistic anti-TIGIT antibodies based on the method used by Joller et al. (27), and then assess the value of agonistic anti-TIGIT antibodies in treating PBC. Obviously, well-designed *in vivo* studies are also needed to validate the efficiency of the CD226 blockade and TIGIT enhancement therapies in PBC.

In addition to proinflammatory T cells, the CD226/TIGIT immune checkpoint also has an important role in modifying the function of Treg cells and dendritic cells (7, 8). For example, TIGIT^+^ Treg cells exhibit a more robust immunosuppressive function and promote the expression of the inhibitory factor IL-10. Furthermore, the expression of TIGIT is correlated with the lineage stability of Treg cells. However, the role CD226 plays in Treg cells is opposite to that of TIGIT. Considering that Treg and dendritic cells are involved in the pathogenesis of autoimmune disease, exploring the balance of the CD226/TIGIT immune checkpoint in these cells is also needed for a deeper understanding of PBC.

There is one limitation of this study should be noted; no histopathological data were obtained owing to the invasive and risky nature of biopsies and the requirements of the ethics committee. Exploring the balance of CD226/TIGIT in infiltrating CD8^+^ T cells from liver lesions and their correlation with the observed alteration of peripheral CD8^+^ T cells may help further elucidate the role this immune checkpoint plays in the pathogenesis of PBC.

In summary, imbalance of the CD226/TIGIT immune checkpoint is involved in the pathogenesis of PBC. The CD226/TIGIT ratio of CD8^+^ T cells is a potential biomarker for evaluating disease status and the prognosis of PBC patients. Moreover, CD8^+^CD226^+^ T cells could serve as a possible therapeutic target and CD226 blocking could inhibit the activity of this cell subset; however, further *in vivo* studies are required to verify these findings.

## Data Availability Statement

The datasets generated for this study are available on request to the corresponding author.

## Ethics Statement

The studies involving human participants were reviewed and approved by The Ethics Committee of the Peking Union Medical College Hospital. The patients/participants provided their written informed consent to participate in this study.

## Author Contributions

YL, CD, and WL contributed conception and design of the study. YF and LW collected the samples. CD, WL, and YC performed the experiment and statistical analysis. CD wrote the first draft of the manuscript. XZ and FZ proofread the manuscript. All authors contributed to the article and approved the submitted version.

## Conflict of Interest

The authors declare that the research was conducted in the absence of any commercial or financial relationships that could be construed as a potential conflict of interest.

## References

[1] Kaplan MM Gershwin ME. Primary biliary cirrhosis. N Engl J Med. (2005) 353:1261–73. 10.1056/NEJMra043898 16177252

[2] Shimoda S Nakamura M Ishibashi H Kawano A Kamihira T Sakamoto N. Molecular mimicry of mitochondrial and nuclear autoantigens in primary biliary cirrhosis. Gastroenterology. (2003) 124:1915–25. 10.1016/S0016-5085(03)00387-1 12806624

[3] Kita H Matsumura S He XS Ansari AA Lian ZX Van de Water J. Quantitative and functional analysis of PDC-E2-specific autoreactive cytotoxic T lymphocytes in primary biliary cirrhosis. J Clin Invest. (2002) 109:1231–40. 10.1172/JCI0214698 11994412 PMC150963

[4] Ronca V Carbone M Bernuzzi F Malinverno F Mousa HS Gershwin ME. From pathogenesis to novel therapies in the treatment of primary biliary cholangitis. Expert Rev Clin Immunol. (2017) 13:1121–31. 10.1080/1744666X.2017.1391093 28994348

[5] Bucktrout SL Bluestone JA Ramsdell F. Recent advances in immunotherapies: from infection and autoimmunity, to cancer, and back again. Genome Med. (2018) 10:79. 10.1186/s13073-018-0588-4 30376867 PMC6208073

[6] Huang C Zhu HX Yao Y Bian ZH Zheng YJ Li L. Immune checkpoint molecules. Possible future therapeutic implications in autoimmune diseases. J Autoimmun. (2019) 104:102333. 10.1016/j.jaut.2019.102333 31564474

[7] Anderson AC Joller N Kuchroo VKDougall WC, Kurtulus S, Smyth MJ, Anderson AC. Lag-3, Tim-3, and TIGIT: Co-inhibitory receptors with specialized functions in immune regulation. Immunity. (2016) 44:989–1004. 10.1016/j.immuni.2016.05.001 27192565 PMC4942846

[8] Dougall WC Kurtulus S Smyth MJ Anderson AC. TIGIT and CD96: new checkpoint receptor targets for cancer immunotherapy. Immunol Rev. (2017) 276:112–20. 10.1111/imr.12518 28258695

[9] Mao L Hou H Wu S Zhou Y Wang J Yu J. TIGIT signalling pathway negatively regulates CD4+ T-cell responses in systemic lupus erythematosus. Immunology. (2017) 151:280–90. 10.1111/imm.12715 28108989 PMC5461105

[10] Luo Q Deng Z Xu C Zeng L Ye J Li X. Elevated expression of immunoreceptor Tyrosine-based inhibitory Motif (TIGIT) on T lymphocytes is correlated with disease activity in Rheumatoid Arthritis. Med Sci Monit. (2017) 23:1232–41. 10.12659/MSM.902454 28282368 PMC5358849

[11] Zhao W Dong Y Wu C Ma Y Jin Y Ji Y. TIGIT overexpression diminishes the function of CD4 T cells and ameliorates the severity of rheumatoid arthritis in mouse models. Exp Cell Res. (2016) 340:132–8. 10.1016/j.yexcr.2015.12.002 26683997

[12] Mosaad YM El-Toraby EE Tawhid ZM Abdelsalam AI Enin AF Hasson AM. Association between CD226 polymorphism and soluble levels in rheumatoid arthritis: Relationship with clinical activity. Immunol Invest. (2018) 47:264–78. 10.1080/08820139.2018.1423570 29319370

[13] Nielsen N Pascal V Fasth AE Sundström Y Galsgaard ED Ahern D. Balance between activating NKG2D, DNAM-1, NKp44 and NKp46 and inhibitory CD94/NKG2A receptors determine natural killer degranulation towards rheumatoid arthritis synovial fibroblasts. Immunology. (2014) 142:581–93. 10.1111/imm.12271 24673109 PMC4107668

[14] Sun C Molineros JE Looger LL Zhou XJ Kim K Okada Y. High-density genotyping of immune-related loci identifies new SLE risk variants in individuals with Asian ancestry. Nat Genet. (2016) 48:323–30. 10.1038/ng.3496 26808113 PMC4767573

[15] Huang Z Fu B Zheng SG Li X Sun R Tian Z. Involvement of CD226+ NK cells in immunopathogenesis of systemic lupus erythematosus. J Immunol. (2011) 186:3421–31. 10.4049/jimmunol.1000569 21296979 PMC3097030

[16] Avouac J Elhai M Tomcik M Ruiz B Friese M Piedavent M. Critical role of the adhesion receptor DNAX accessory molecule-1 (DNAM-1) in the development of inflammation-driven dermal fibrosis in a mouse model of systemic sclerosis. Ann Rheum Dis. (2013) 72:1089–98. 10.1136/annrheumdis-2012-201759 23161903

[17] Abu El-Ella SS Khattab ESAEH El-Mekkawy MS El-Shamy AA. CD226 gene polymorphism (rs763361 C>T) is associated with susceptibility to type 1 diabetes mellitus among Egyptian children. Arch Pediatr. (2018) 25:378–82. 10.1016/j.arcped.2018.06.009 30145014

[18] Heathcote EJ. Management of primary biliary cirrhosis. The American association for the study of liver diseases practice guidelines. Hepatology. (2000) 31:1005–13. 10.1053/he.2000.5984 10733559

[19] Hosseini A Gharibi T Marofi F Babaloo Z Baradaran B. CTLA-4: from mechanism to autoimmune therapy. Int Immunopharmacol. (2020) 80:106221. 10.1016/j.intimp.2020.106221 32007707

[20] Bowlus CL Yang GX Liu CH Johnson CR Dhaliwal SS Frank D. Therapeutic trials of biologics in primary biliary cholangitis: an open label study of abatacept and review of the literature. J Autoimmun. (2019) 101:26–34. 10.1016/j.jaut.2019.04.005 31027870

[21] Andrews LP Yano H Vignali DAA Inhibitory receptors and ligands beyond PD-1, PD-L1 and CTLA-4: breakthroughs or backups. Nat Immunol. (2019) 20:1425–34. 10.1038/s41590-019-0512-0 31611702

[22] Qin S Xu L Yi M Yu S Wu K Luo S. Novel immune checkpoint targets: moving beyond PD-1 and CTLA-4. Mol Cancer. (2019) 18:155. 10.1186/s12943-019-1091-2 31690319 PMC6833286

[23] Yang GX Lian ZX Chuang YH Moritoki Y Lan RY Wakabayashi K. Adoptive transfer of CD8(+) T cells from transforming growth factor beta receptor type II (dominant negative form) induces autoimmune cholangitis in mice. Hepatology. (2008) 47:1974–82. 10.1002/hep.22226 18452147 PMC2749317

[24] Betts MR Koup RA. Detection of T-cell degranulation: CD107a and b. Methods Cell Biol. (2004) 75:497–512. 10.1016/S0091-679X(04)75020-7 15603439

[25] Aktas E Kucuksezer UC Bilgic S Erten G Deniz G. Relationship between CD107a expression and cytotoxic activity. Cell Immunol. (2008) 254:149–54. 10.1016/j.cellimm.2008.08.007 18835598

[26] Dixon KO Schorer M Nevin J Etminan Y Amoozgar Z Kondo T. Functional anti-TIGIT antibodies regulate development of autoimmunity and antitumor immunity. J Immunol. (2018) 200:3000–7. 10.4049/jimmunol.1700407 29500245 PMC5893394

[27] Joller N Hafler JP Brynedal B Kassam N Spoerl S Levin SD. Cutting edge: TIGIT has T cell-intrinsic inhibitory functions. J Immunol. (2011) 186:1338–42. 10.4049/jimmunol.1003081 21199897 PMC3128994

